# Tiger Amulet inspired high-security holographic encryption via liquid crystals

**DOI:** 10.1515/nanoph-2023-0040

**Published:** 2023-03-15

**Authors:** Xianjing Huang, Dong Zhu, Zhou Zhou, Kuixian Chen, Guoxing Zheng, Peng Chen, Yan-Qing Lu, Zile Li

**Affiliations:** Electronic Information School, Wuhan University, Wuhan 430072, China; National Laboratory of Solid State Microstructures, Key Laboratory of Intelligent Optical Sensing and Manipulation, and College of Engineering and Applied Sciences, Nanjing University, Nanjing 210023, China; Peng Cheng Laboratory, Shenzhen 518055, China; Wuhan Institute of Quantum Technology, Wuhan 430206, China

**Keywords:** encryption, hologram, liquid crystal

## Abstract

Due to the precise and continuous regulation of phase, holographic encryption based on metasurfaces and liquid crystals (LCs) has been proposed to encrypt the information by manipulating the wavelength, polarization, etc. However, the security cannot be fully guaranteed since the requirements of decoding methods for these schemes are generally not very strict and vulnerable for exhaustive attack. Furthermore, any part of the hologram stolen may lead to the disclosure of the hidden information regardless of the generation mode of phase delay or the selection of media material, so the security needs to be further improved. Here, inspired by Tiger Amulet, embodying the encryption consciousness of ancient China, we propose a simple but effective encryption method and design a “four-in-one” hologram based on photopatterned LCs. Specifically, the most important encrypted image can only be displayed when the four LC holograms in the same group are spliced into a whole according to the designed order. On the contrary, the camouflage information would be displayed if the holograms are placed in the optical path separately or spliced in wrong order. It is even more interesting that with the LC directors tilted with applied external voltages, the holographic efficiency of the LC hologram will change accordingly. This sets further demanding requirement on the decryption condition and thus increases the encryption security. With the advantages of simple design, high security, and low crosstalk, our encryption scheme has great potential in the fields of information hiding and image encryption.

## 1 Introduction

With the development of information technology, people are exchanging information more and more frequently. In this context, the issue of information security has received more attention than ever before, and amounts of efforts are put in exploring new encryption technology to enhance the information security. Among numerous technical methods, optical holographic encryption, as a new encryption approach, has become one of the most important research contents of modern encryption technology due to its unique technical characteristics [1, 2]. In optical holographic encryption, information can be hidden in a variety of parameters, such as phase, spatial frequency, polarization or wavelength, which empowers the optical encryption excellent coding capacity and flexible key design.

In recent years, the research of metasurfaces based on Pancharatnam–Berry (PB) phase has attracted a lot of attention. Due to its precise and continuous phase regulation without increasing the processing complexity, it enables the reconstruction of high-quality holographic images [3–7]. Thus, meta-holograms are increasingly used in the field of optical encryption [8–12]. Many feasible methods are to encrypt the information by manipulating the wavelength [13, 14], polarization [15–18], incidence angle [19], topological charge [20, 21], observation spaces [22–24] or spatial frequency range [25] and so on. Meanwhile, liquid crystals (LCs) have also been widely used in spatial light modulations owing to the unique optical characteristics of polarization control and sensitivity to external stimuli response. Designed LCs and meta-holograms can be combined to realize stimuli-responsive dynamic displays [26–28]. Thus, LC-integrated metasurfaces can also work as a candidate for information encryption, which can be implemented by changing the applied voltages [29, 30] or environmental temperature [31]. Recently, researchers have found that LC can control the PB phase of light waves like metasurface, and holographic encryption based on LCs has been proposed [32–34]. Compared to meta-hologram, LC holographic encryption has the advantages of adjustable spectral response, large-area manufacturing, high efficiency, low cost, dynamic switching ability, easy cutting and splicing, and compatibility with existing commercial LCD devices [35–37]. However, the security cannot be fully guaranteed since the requirements of decoding methods for these schemes are generally not very strict to some extent. It is still possible to get the hidden information in the case of wavelength offset in a certain range, polarization state with an error and less accurate incidence angle, etc. Even if the information may not be as clear and accurate, it is not far from complete decryption. Furthermore, because any part of the hologram designed by the traditional algorithms contains the hidden information, any part stolen may lead to the disclosure of the hidden information regardless of the generation mode of phase delay (geometric phase, transmission phase, etc.) or the selection of media material (metasurfaces, LCs). Thus, the security needs to be further improved.

**Figure 1:**
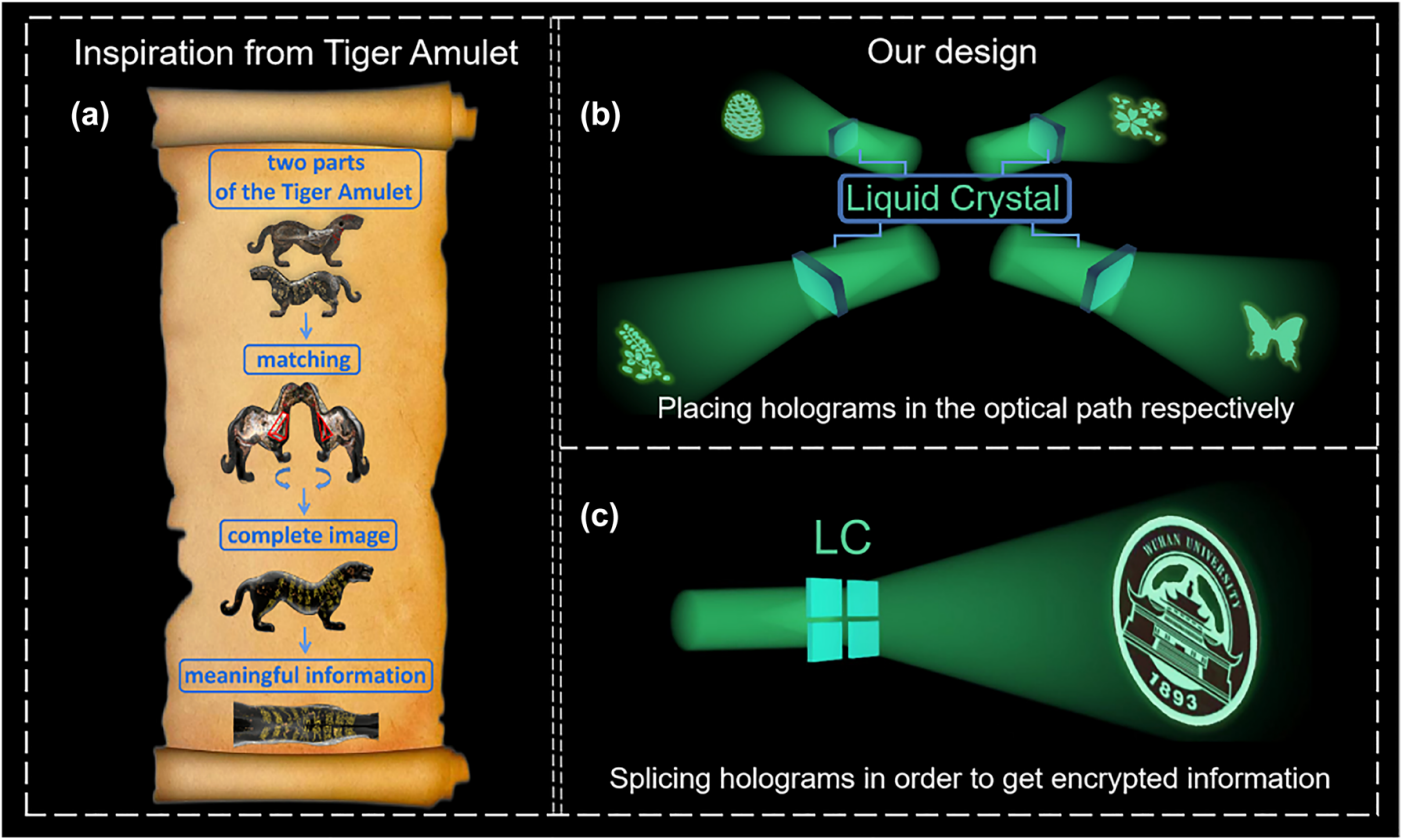
Inspiration and operation schematics of the “four-in-one” LC hologram. (a) The inspiration from Tiger Amulet. (b) Schematic illustration of the reconstruction result when four small holograms are placed in the optical path to work separately. (c) Schematic illustration of the reconstruction result when four small holograms are spliced into a whole.

Inspired by Tiger Amulet, a tiger-shaped tally issued to generals as imperial authorization for troop movement in ancient China, we propose a high-security encryption method based on “four-in-one” LC hologram to address the problem of information disclosure in holographic encryption. The basic idea of how the Tiger Amulet works is schematically demonstrated in Figure 1(a). It could be seen that only the Tiger Amulet in the same group can be combined to form a complete pattern and plays the role of conveying orders, while the forged Tiger Amulet cannot be closely matched with the real one to show the real meaningful information. Our encryption method works in a similar logic: as shown in Figure 1(c), the most important encrypted image can only be displayed when the four LC holograms in the same group are spliced into a whole according to the designed order; when they are spliced in the wrong order or placed in the optical path respectively, camouflage information instead of actually encrypted information would be decoded, as seen in Figure 1(b). With many advantages such as large-area manufacturing and low cost, LC is chosen as the media material to implement our new encryption method. Moreover, by applying external voltages, the out-of-plane orientation angles of LC director will vary [38], leading to different holographic efficiency and more demanding decryption condition. Therefore, our method provides an encryption scheme with simple design, high security, and low crosstalk, which has great potential in the fields of information hiding and image encryption.

## 2 Design of the LC-based four-in-one holograms

LC is an anisotropic medium. It is possible to modulate phase continuously in LCs through PB phase due to the spin orbit interaction of light in heterogeneous anisotropic media [39–41]. An LC director has two orientation angles: an in-plane orientation angle *θ* and an out-of-plane orientation angle *α*. We design all working unit-cells of LC device with the same size, but different in-plane orientation angles *θ* (−90°–90°). Here, we control the PB phase by varying the in-plane orientation angles unit by unit, and the PB phase is exactly twice the in-plane orientation angle.

**Figure 2:**
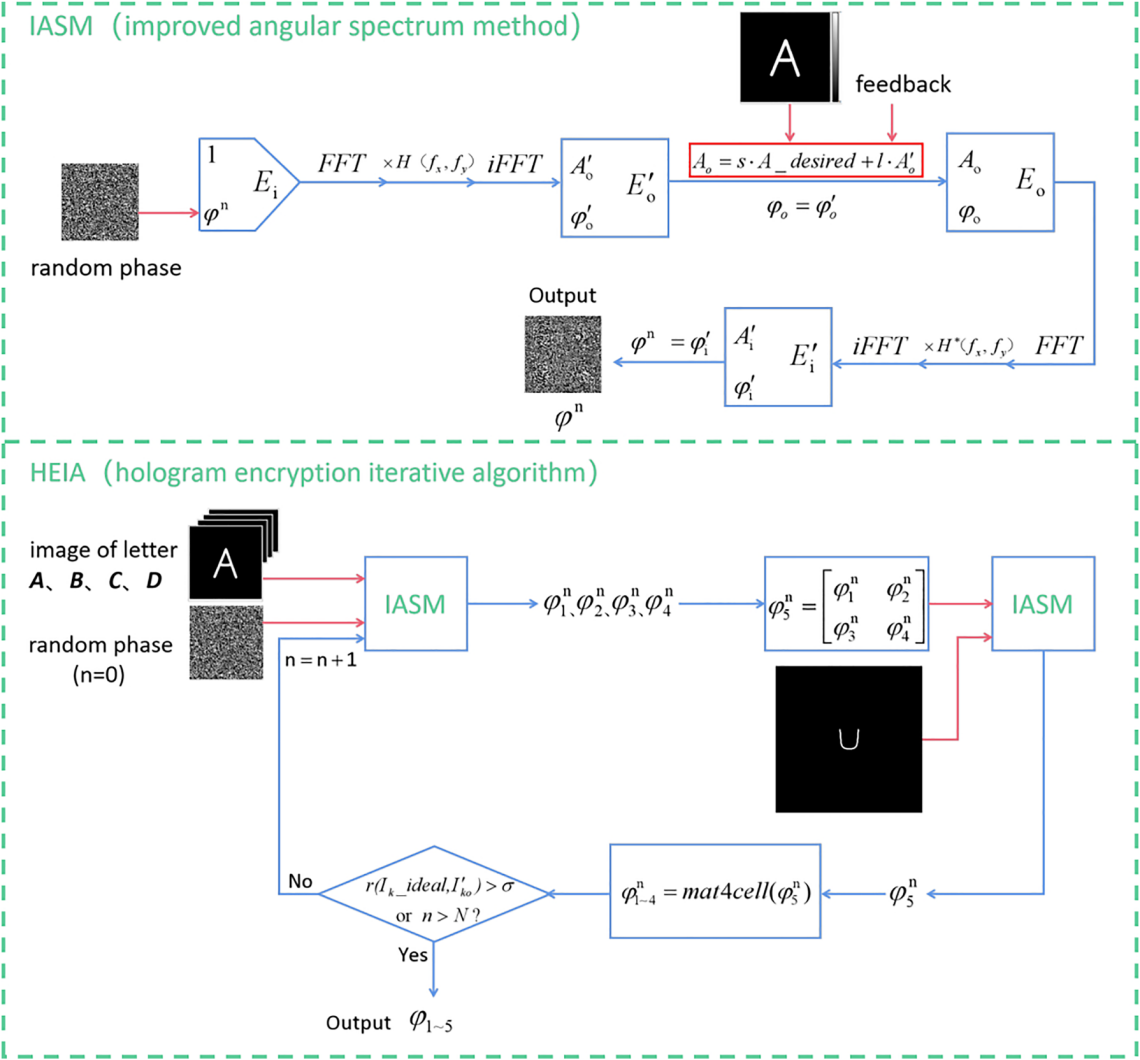
Flowchart of the improved angular spectrum method and hologram encryption iterative algorithm. “FFT” and “iFFT” represent fast Fourier transformation and inverse fast Fourier transformation, respectively. “H” represents angular spectrum propagation function and “H*” represents its conjugate.

It is known that separate small holograms can be designed to display different holographic images. Benefiting from the degree of freedom in the holographic design, it is possible to display another holographic image when the phase matrices of separate small holograms are combined into the phase matrix of a large hologram according to a designed order. Based on this general idea, we propose a hologram encryption iterative algorithm (HEIA) to calculate the phase distribution of LC hologram to realize the “four-in-one” holographic encryption. In our design, the overall iterative optimization of five images is carried out, and the correlation coefficient is used to evaluate the quality of the reconstructed image. In each iteration, we also introduce different weight information to different images to adjust the reconstruction quality of images with different sizes. By carefully designing the weighted value of the amplitude of the desired output light field and that in the calculated result, high reconstruction quality of all the five images can be achieved. For the optimization of each separate holograms, an improved angular spectrum method (IASM) is utilized, and the flow chart is shown in Figure 2. Different from its traditional counterpart, in this method the amplitude of the updated output light field is made up of the weighted value of the desired and calculated light field amplitude, which replaces the preceding one obtained by angular spectrum diffraction. By following this process, the diffraction result of each iteration will be superimposed on the output light field information. After inverse diffraction, it will return to the input light field as feedback, so as to accelerate the iteration speed [42].

After four holographic images (*A*, *B*, *C*, *D*) and random phase are put into the IASM in turn, we will get the four matrices of phase information for the current iteration. After that we combine four small phase matrices into the phase matrix of the large hologram. When putting it and the encrypted holographic image (*U*) into IASM and then getting the new large phase matrix, the phase matrix of the large hologram is decomposed into four small phase matrices and one iteration process is completed. Finally, the iterative process for the whole is repeated until the correlation coefficient between the desired light intensity distribution and that calculated by diffraction theory is greater than the preset threshold *σ* or the number of iterations *n* reaches *N* (the maximum number of iterations). In the end, we get the phases of the designed hologram.

## 3 Experimental demonstration of the LC-based holographic encryption

Based on the hologram encryption iterative algorithm, we design an LC device to realize the display of different patterns of four small LC holograms separately and combined. The four small LC holograms are composed of 124 × 124 pixels, and four different letter images (“*A*”, “*B*”, “*C*”, and “*D*”) are selected as the target images. Another letter image (“*U*”) is selected as the target image for the large hologram.

The fabrication process of the LC sample can be mainly divided into two parts: one is the preparation of the LC empty cell, and the other is the photopatterning technology to realize the designed in-plane LC orientations. First, ITO glass substrates were spin-coated with the UV-polarization-sensitive azo-dye SD1 layer, and then covered with the other substrate to form the empty cell with 8 μm spacers. Second, the DMD-based dynamic photopatterning system is used to implement the multistep partly overlapping exposure process [37, 43]. The designed in-plane director distributions can be obtained in nematic LCs, forming the LC hologram.

**Figure 3:**
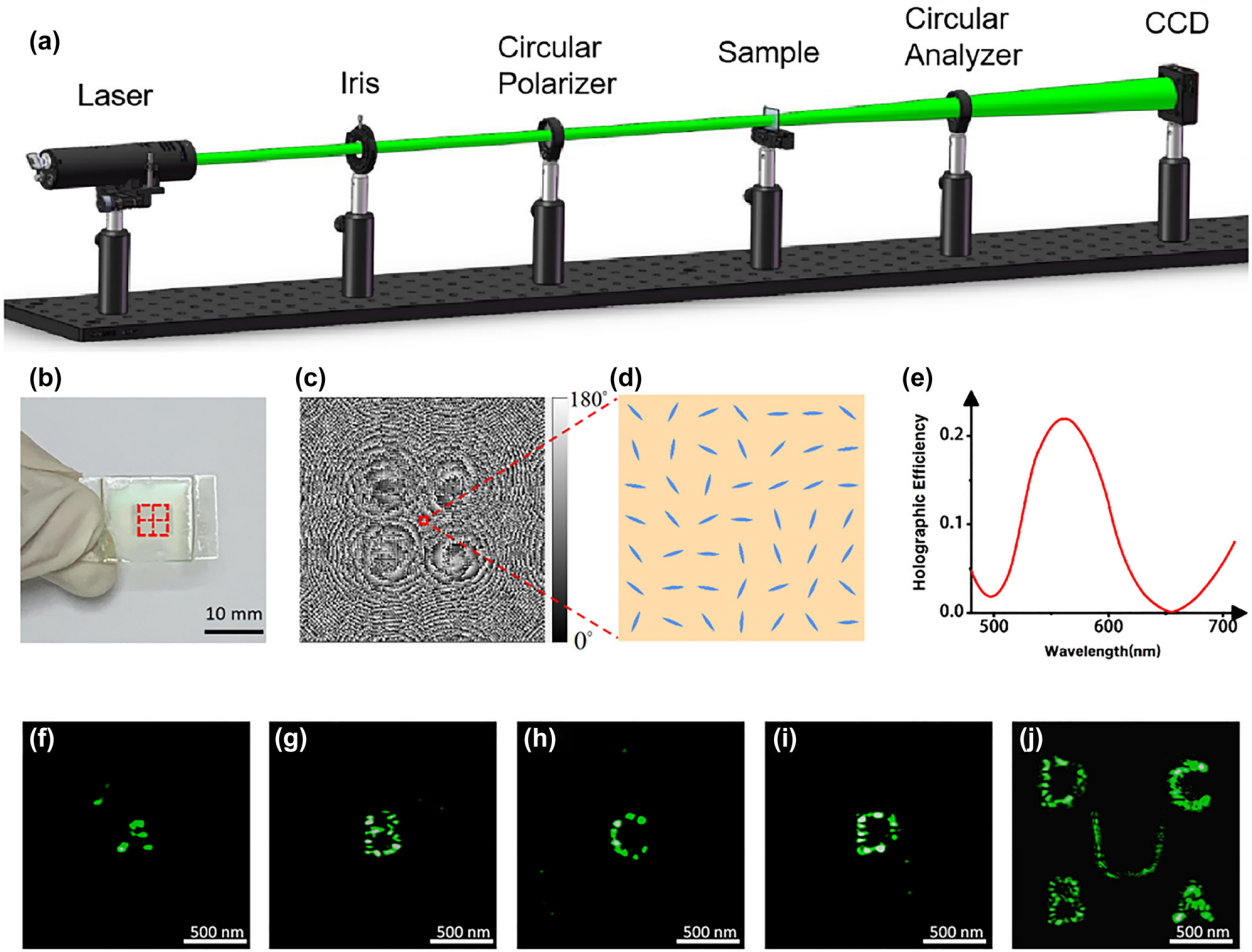
Experimental results of the “four-in-one” LC hologram without applied voltage. (a) Optical setup for observing holographic image. (b) The photo of the “four-in-one” LC hologram and the marked red box indicates the working area. (c) The final in-plane orientation angle distribution. (d) Schematic of zoom-in view of the in-plane orientation angle distribution. (e) Holographic efficiency of the LC device for different wavelengths without applied external voltage. (f)–(j) The captured holographic images of 4 small holographic images and the combined big one at the wavelength of 560 nm.

To characterize its performance, the sample is placed in an optical path as shown in Figure 3(a). A super-continuum laser (YSL SC-pro) is used to illuminate the sample. The incident laser beam is converted into circularly polarized (CP) light after passing through the circular polarizer. And a circular analyzer is introduced as an optical filter to make sure all the light that can pass through is cross-polarized CP light. All holographic images were captured by a charge coupled device (CCD).

Figure 3(b) exhibits the LC device composed of 248 × 248 effective working units, which corresponds to the in-plane orientation angles distribution shown in Figure 3(c). In our experiment, the LC cell is 8 μm thick with each working unit-cell measuring 20.7 × 20.7 μm^2^. We choose 560 nm as the design wavelength. Figure 3(f)–(i) shows the holographic images obtained when the four small holograms are illuminated, respectively, from which it could be seen that all the letters are reconstructed clearly. And the encrypted information, i.e., pattern “*U*”, could be obtained when the four small holograms are illuminated simultaneously in the right order, which demonstrates the effectiveness of our encryption method. We also measure holographic efficiency, the ratio of the transmitted energy reconstructing the holographic image to the incident light energy, without applied external voltage. Figure 3(e) shows the variant holographic efficiency in the wavelength range from 480 to 710 nm. At the operation wavelength of 560 nm, holographic efficiency reaches its maximum of 22.7%. Moreover, to judge the quality of the holographic images in this work, we use a more intuitive parameter, correlation coefficient [12], to compare the similarity between the experimentally captured images and the target images. The correlations between the captured holographic images (Figure 3(f)–(j)) and the corresponding target images are 0.6978, 0.6863, 0.6802, 0.6776, and 0.6722 respectively. The correlation coefficients for 5 holographic images are higher than 0.67, indicating that the experimental results are in good agreement with the design.

**Figure 4:**
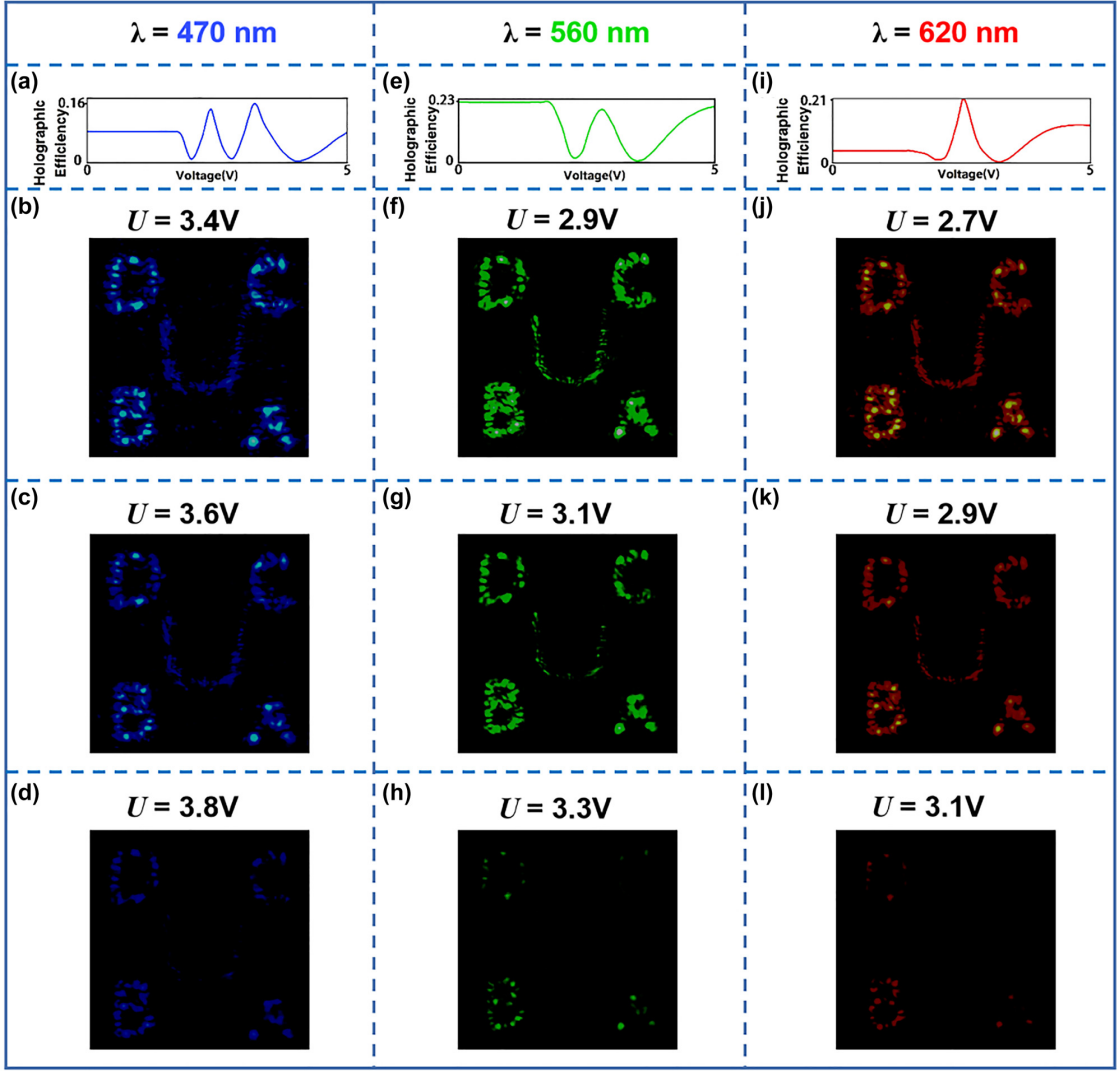
Spectral response of the “four-in-one” LC hologram with different external electric field. (a), (e), and (i) Holographic efficiency for different applied voltages. The operating wavelength is (a) 470 nm, (e) 560 nm, (i) 620 nm. (b)–(d), (f)–(h), and (j)–(l) Holographic images under different applied voltages at the wavelength of (b)–(d) 470 nm, (f)–(h) 560 nm, and (j)–(l) 620 nm.

To investigate the broadband response of our sample with different applied voltages, we alter the wavelength of the super-continuum laser source from 470 nm to 620 nm and employ a commercial signal generator to provide an external electric field. Figure 4(a), (e), and (i) shows the experimental results of the holographic efficiency at respective wavelength under the applied voltage ranging from 0 to 5 V. We can see that there is a threshold voltage which means when the voltage does not reach the threshold, the holographic efficiency will not change. Then, the increasing voltage leads to the change of holographic efficiency, which indicates the latent capacity of tunable spectral response. As shown in Figure 4(b)–(d), (f)–(h), and (j)–(l), when choosing wavelengths of 470, 560, and 620 nm, holographic images with different brightness could be obtained under different applied voltages. Moreover, as for a certain operating wavelength, the holographic efficiency would decrease from the maximum to almost zero within a specific applied voltage range, which suggests that we could apply this property to the encryption process.

## 4 Discussions

**Figure 5:**
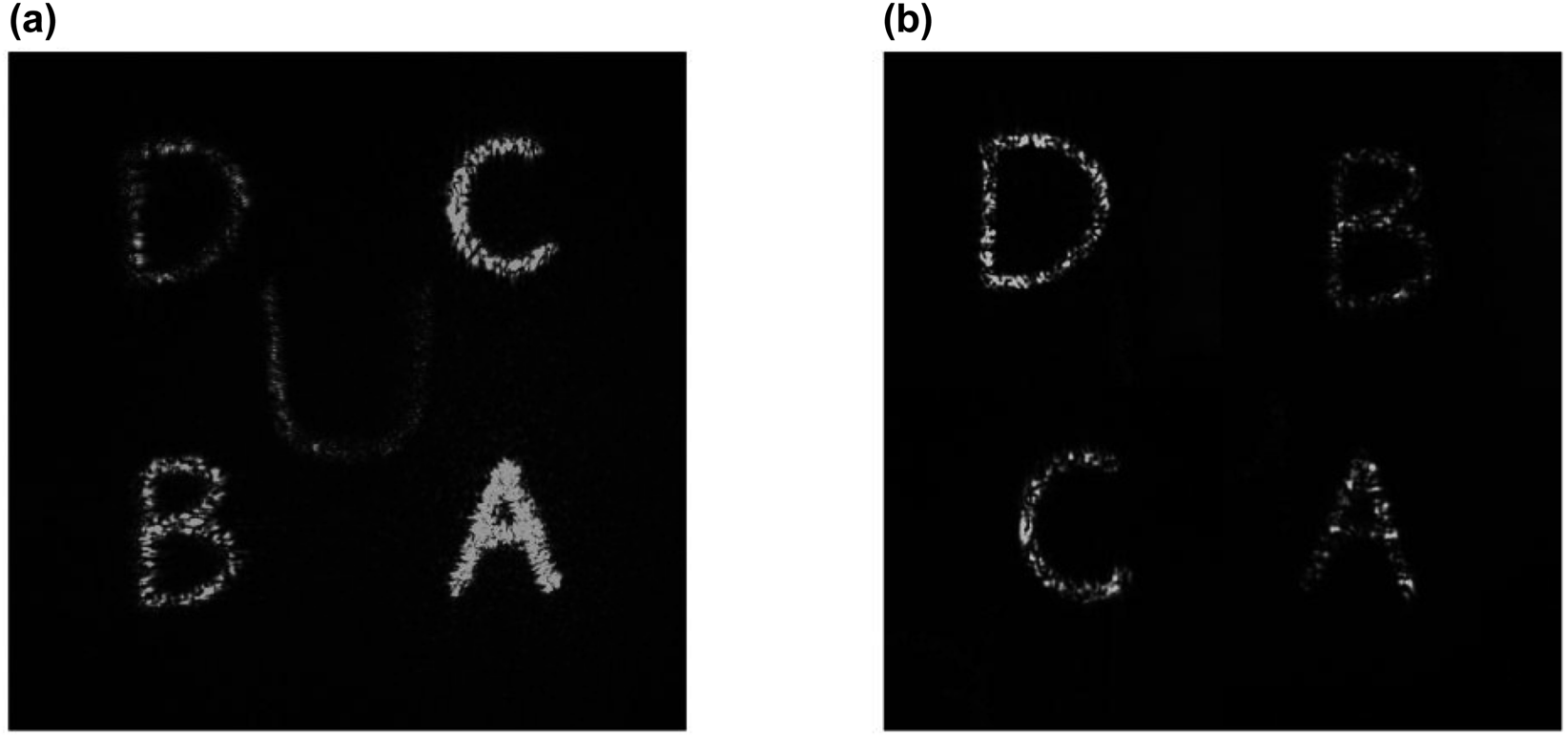
Experimental results of the “four-in-one” LC hologram in different orders. (a) Holographic image of four designed holograms in the right order. (b) Holographic image of four jointly designed holograms in the wrong order.

In addition to the above experiments, the security of our proposed encryption method is further verified through splicing holograms in different orders, and the results are shown in Figure 5. When four small holograms are jointly designed and spliced in the right order, we can see the letter “*U*” in the center of holographic image; but when the arrangement order is inconsistent with the designed one, it could be seen from Figure 5(b) that the letter “*U*” cannot be obtained. Therefore, our design can be used as an encryption device for authentication just like Tiger Amulet. Higher security performance will be obtained if using 6, 8, or even more LC holograms. However, the computing time of the holographic encryption algorithm will increase greatly at the same time. And the setup alignment requirements will become stricter to obtain encrypted information after splicing all the small holograms and the quality of the reconstructed image will also be affected. There is a trade-off when choosing schemes with different number of the LC holograms.

Our core idea in this work is “multiple in one” correct matching to display correct information, which is inspired by Tiger Amulet. Using this idea and the optimization algorithm proposed in the paper, our design has a further demanding requirement on the decryption condition and thus increases the encryption security regardless of the generation mode of phase delay (geometric phase, transmission phase, etc.) or the selection of media material (metasurfaces, LCs). As a result, the scheme used in our work is free from the problem in previous holographic encryption methods, i.e., any part of the hologram stolen may lead to the disclosure of the hidden information. In a word, the Tiger Amulet technique is used to address the problem of information disclosure in holographic encryption. Overall, the proposed LC hologram encryption scheme has many technical advantages compared to previous solutions. First, in our design, the encrypted image is hidden when the four small holograms work separately in the optical path. But when they are correctly combined, the encrypted image could be decoded and displayed. This breaks through the property that any part of the traditional hologram contains whole object light wave information. So even if a single small hologram is stolen here, the encrypted information cannot be recovered, and only the wrong information as camouflage would be decoded, which increases the information security and mitigate the problem of information disclosure in holographic encryption. Second, benefitting from the electrical tunability of LC materials, i.e., each LC director can tilt from the state of in-plane to out-of-plane when applied external voltages, the encryption safety based on the proposed “four-in-one” LC hologram can be further enhanced. The applied external voltage of the four small holograms can be designed with different value independently, which allows the setting of more demanding conditions to get the encrypted message and reduces the risk of information disclosure. Last but not least, compared with holograms based on metasurfaces, our LC hologram is more practical in terms of large-area manufacturing. Considering current mature technology in LC display industry, our design is simple to implement and it is possible to realize the mass production of the proposed encryption device for practical applications.

## 5 Conclusions

In this paper, we propose and experimentally demonstrate an encryption method based on the LC hologram. Only when the four holograms in the same group are placed in the optical path at the same time and in the designed order, can the most important encrypted images be displayed. As a contrast, other camouflage images would be displayed when the small holograms work separately in the optical path. Due to the introduction of iterative optimization and the weighted value of the amplitude based on the traditional angular spectrum theory, a new encryption method based on hologram splicing is proposed and demonstrated. Since the observed holographic image would vary as the applied external voltages of the LC hologram changes, the applied electrical signal can also be considered in the encryption process, leading to enhanced security of the encrypted information. And the mature LC display technology makes our design more practical and adaptable. Overall, with these unique characteristics of simple design, high security, low crosstalk, and large-area manufacturing, our proposed LC device has great potential in various applications such as information hiding, image encryption, and so on.

## References

[1] Javidi B., Nomura T. (2000). Securing information by use of digital holography. *Opt. Lett.*.

[2] Fang X., Ren H., Gu M. (2020). Orbital angular momentum holography for high-security encryption. *Nat. Photonics*.

[3] Khorasaninejad M., Crozier K. B. (2014). Silicon nanofin grating as a miniature chirality-distinguishing beam-splitter. *Nat. Commun.*.

[4] Zheng G., Mühlenbernd H., Kenney M., Li G., Zentgraf T., Zhang S. (2015). Metasurface holograms reaching 80% efficiency. *Nat. Nanotechnol.*.

[5] Li Z., Zheng G., He P. (2015). All-silicon nanorod-based Dammann gratings. *Opt. Lett.*.

[6] Khorasaninejad M., Chen W., Devlin R. C., Oh J., Zhu A., Capasso F. (2016). Metalenses at visible wavelengths: diffraction-limited focusing and subwavelength resolution imaging. *Science*.

[7] Mou Z., Zhou C., Lu P., Yue Q., Wang S., Teng S. (2021). Structured vortices generated by metasurface holography. *Photon. Res.*.

[8] Dai Q., Guan Z., Chang S. (2020). A single-celled tri-functional metasurface enabled with triple manipulations of light. *Adv. Funct. Mater.*.

[9] Deng L., Deng J., Guan Z. (2020). Malus-metasurface-assisted polarization multiplexing. *Light Sci. Appl.*.

[10] Liang C., Deng L., Dai Q. (2021). Single-celled multifunctional metasurfaces merging structural-color nanoprinting and holography. *Opt. Express*.

[11] Fu R., Shan X., Deng L. (2021). Multiplexing meta-hologram with separate control of amplitude and phase. *Opt. Express*.

[12] Zhou Z., Wang Y., Chen C. (2022). Holographic meta-image displays enabled by dual-degeneracy. *Small*.

[13] Ye W., Zeuner F., Li X. (2016). Spin and wavelength multiplexed nonlinear metasurface holography. *Nat. Commun.*.

[14] Jin L., Dong Z., Mei S. (2018). Noninterleaved metasurface for (26-1) spin-and wavelength-encoded holograms. *Nano Lett*..

[15] Liu H. C., Yang B., Guo Q. (2017). Single-pixel computational ghost imaging with helicity-dependent metasurface hologram. *Sci. Adv.*.

[16] Li Z., Chen C., Guan Z. (2020). Three-channel metasurfaces for simultaneous meta-holography and meta-nanoprinting: a single-cell design approach. *Laser Photonics Rev.*.

[17] Shan X., Li Z., Dai Q. (2021). Metasurfaces with single-sized antennas for reconstructing full-color holographic images without cross talk. *Opt. Lett.*.

[18] Zhang S., Huang L., Geng G., Li J., Li X., Wang Y. (2022). Full-Stokes polarization transformations and time sequence metasurface holographic display. *Photon. Res.*.

[19] Li X., Chen L., Li Y. (2016). Multicolor 3D meta-holography by broadband plasmonic modulation. *Sci. Adv.*.

[20] Jin L., Huang Y. W., Jin Z. (2019). Dielectric multi-momentum meta-transformer in the visible. *Nat. Commun.*.

[21] Ren H., Fang X., Jang J., Bürger J., Rho J., Maier S. A. (2020). Complex-amplitude metasurface-based orbital angular momentum holography in momentum space. *Nat. Nanotechnol.*.

[22] Huang K., Dong Z., Mei S. (2016). Silicon multi-meta-holograms for the broadband visible light. *Laser Photonics Rev.*.

[23] Wei Q., Huang L., Li X., Liu J., Wang Y. (2017). Broadband multiplane holography based on plasmonic metasurface. *Adv. Opt. Mater.*.

[24] Liang X., Deng L., Shan X. (2021). Asymmetric hologram with a single-size nanostructured metasurface. *Opt. Express*.

[25] Deng J., Yang Y., Tao J. (2019). Spatial frequency multiplexed meta-holography and meta-nanoprinting. *ACS Nano*.

[26] Naveed M. A., Kim J., Javed I. (2022). Novel spin-decoupling strategy in liquid crystal-integrated metasurfaces for interactive metadisplays. *Adv. Opt. Mater.*.

[27] Kim I., Ansari M. A., Mehmood M. Q. (2020). Stimuli-responsive dynamic metaholographic displays with designer liquid crystal modulators. *Adv. Mater.*.

[28] Ni Y., Chen C., Wen S., Xue X., Sun L., Yang Y. (2022). Computational spectropolarimetry with a tunable liquid crystal metasurface. *eLight*.

[29] Li J., Yu P., Zhang S., Liu N. (2020). Electrically-controlled digital metasurface device for light projection displays. *Nat. Commun.*.

[30] Kim I., Jang J., Kim G. (2021). Pixelated bifunctional metasurface-driven dynamic vectorial holographic color prints for photonic security platform. *Nat. Commun.*.

[31] Choi C., Mun S. E., Sung J., Choi K., Lee S. Y., Lee B. (2021). Hybrid state engineering of phase-change metasurface for all-optical cryptography. *Adv. Funct. Mater.*.

[32] Chen P., Wei B. Y., Hu W., Lu Y. Q. (2020). Liquid-crystal-mediated geometric phase: from transmissive to broadband reflective planar optics. *Adv. Mater.*.

[33] Chen P., Ma L. L., Hu W. (2019). Chirality invertible superstructure mediated active planar optics. *Nat. Commun.*.

[34] Li Z. X., Ruan Y. P., Chen P. (2021). Liquid crystal devices for vector vortex beams manipulation and quantum information applications. *Chin. Opt. Lett.*.

[35] Xiong J., Wu S. T. (2021). Planar liquid crystal polarization optics for augmented reality and virtual reality: from fundamentals to applications. *eLight*.

[36] Chen K., Xu C., Zhou Z. (2022). Multifunctional liquid crystal device for grayscale pattern display and holography with tunable spectral-response. *Laser Photonics Rev.*.

[37] Chen P., Shen Z. X., Xu C. T. (2022). Simultaneous realization of dynamic and hybrid multiplexed holography via light-activated chiral superstructures. *Laser Photonics Rev.*.

[38] Wang C. T., Tam A., Meng C., Tseng M. C., Li G., Kwok H. S. (2020). Voltage-controlled liquid crystal Pancharatnam-Berry phase lens with broadband operation and high photo-stability. *Opt. Lett.*.

[39] Li Z., Yu S., Zheng G. (2020). Advances in exploiting the degrees of freedom in nanostructured metasurface design: from 1 to 3 to more. *Nanophotonics*.

[40] Berry M. (1984). Quantal phase factors accompanying adiabatic changes. *Proc. Roy. Soc. Lond. Math. Phys. Sci.*.

[41] Zhu L., Xu C. T., Chen P. (2022). Pancharatnam-Berry phase reversal via opposite-chirality-coexisted superstructures. *Light Sci. Appl.*.

[42] Liu H., Ji Y. (2013). An ameliorated fast phase retrieval iterative algorithm based on the angular spectrum theory. *Acta Phys. Sin.*.

[43] Chen P., Ma L. L., Duan W. (2018). Digitalizing self-assembled chiral superstructures for optical vortex processing. *Adv. Mater.*.

